# Mitochondrial network expansion and dynamic redistribution during islet morphogenesis in zebrafish larvae

**DOI:** 10.1002/1873-3468.14508

**Published:** 2022-10-19

**Authors:** Julia Freudenblum, Dirk Meyer, Robin A. Kimmel

**Affiliations:** ^1^ Institute of Molecular Biology/CMBI University of Innsbruck Austria

**Keywords:** image analysis, *in vivo* imaging, islet, mitochondria, pancreas, zebrafish

## Abstract

Mitochondria, organelles critical for energy production, modify their shape and location in response to developmental state and metabolic demands. Mitochondria are altered in diabetes, but the mechanistic basis is poorly defined, due to difficulties in assessing mitochondria within an intact organism. Here, we use *in vivo* imaging in transparent zebrafish larvae to demonstrate filamentous, interconnected mitochondrial networks within islet cells. Mitochondrial movements highly resemble what has been reported for human islet cells *in vitro*, showing conservation in behaviour across species and cellular context. During islet development, mitochondrial content increases with emergence of cell motility, and mitochondria disperse within fine protrusions. Overall, this work presents quantitative analysis of mitochondria within their native environment and provides insights into mitochondrial behaviour during organogenesis.

## Graphical abstract

Mitochondria, the cell's energy‐producing organelle, form a dynamic network which responds to developmental and metabolic status. They are important for pancreatic beta cell function, and their dysfunction contributes to diabetes. In order to better understand the coordinated cellular and mitochondrial dynamics which accompany islet development and diabetes, we developed methods to monitor mitochondria in single islet cells within the intact zebrafish larva.

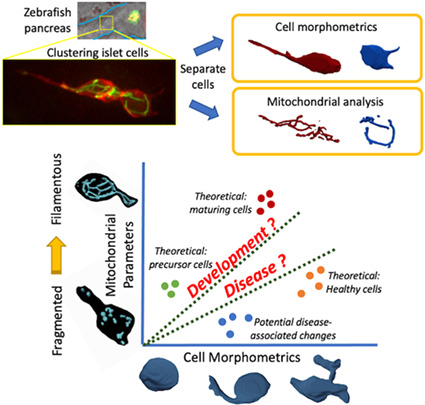

## Abbreviations

**2D**, two‐dimensional

**3D**, three‐dimensional

**dpf**, days post‐fertilisation

**GFP**, green fluorescent protein

**ins**, insulin

**MIP**, maximum intensity projection

**ROS**, reactive oxygen species

Energy‐producing mitochondria display complex and dynamic networks, with a close relation between mitochondria shape and function. Mitochondria vary in position and morphology in response to differentiated state of the cell and during cell migration [1, 2]. In pancreatic beta‐cells, mitochondria serve a critical additional function in regulating the insulin‐secretion response [3]. In response to changing environment and metabolic conditions, mitochondria assume shapes ranging from small discrete organelles to large interconnected networks [4]. Fusion of mitochondria serves to mix mitochondrial contents, and formation of elongated structures increases ATP production which helps mitochondria to avoid mitophagy under conditions of stress [2, 4]. Fission, the breaking down of the mitochondria network into smaller units, permits mitochondrial movement to subcellular locations with enhanced energy requirements, and promotes removal of defective units by mitophagy [2, 5].

Descriptions of mitochondrial morphology and dynamics have until recently been largely qualitative, with application of subjective categorisations [6]. Along with advances in imaging techniques, quantitative methods for defining mitochondrial features have been developed [7]. Newer approaches integrate machine learning algorithms, high‐throughput image acquisition and automation of analysis [8, 9]. Methods have been primarily developed for two‐dimensional images, which can in some cases be applied to images of three‐dimensional samples [7, 10, 11, 12]. Despite these advances, analysis of mitochondrial networks has predominantly focused on cells in culture and there have until now been few studies of mitochondrial dynamics in whole tissue.

In model organisms it is possible to image mitochondria in their native environment, during development and in disease models [13, 14]. The transparent zebrafish offers accessibility for *in vivo* imaging in a vertebrate system that is highly similar to mammals in genetics, development and physiology. Tissue specific mitochondria labelling has been used to characterise mitochondrial transport in neurons [14, 15], and mitochondrial network maturation during somite development [16].

As mitochondria are dynamic structures forming complex networks, they are best studied in an intact model organism in which three‐dimensional visualisation of these structures over time can be achieved. In this work, we use the zebrafish as a vertebrate model system in which pancreatic islet mitochondria can be fluorescently labelled and their morphology observed, during developmental processes and pathophysiological manipulation. By combining a vertebrate model with high‐resolution four‐dimensional imaging and open source analytic tools, we achieve semi‐automated quantitative analysis of mitochondrial morphology, which can be applied to clarify relationships between mitochondrial dynamics, organ morphogenesis, and cellular and organismal physiology. We show that islet cell mitochondria *in vivo* recapitulate the highly networked morphology and dynamic behaviours seen in cell culture, and that the mitochondrial network expands not only in response to oxidative stress, but also during morphogenesis, providing metabolic support in regions of high cellular motility.

## Materials and methods

### Zebrafish maintenance and transgenic fish lines

Zebrafish (*Danio rerio*) were maintained according to standard protocols [17]. The *neurod*:*mitoGFP* construct (generously provided by Alex Nechiporuk, Oregon Health & Science University, USA) contains the mitochondrial localisation sequence from cytochrome oxidase VIII fused to the N terminus of eGFP [18, 19]. Plasmid DNA containing Tol2 sites was co‐injected with *transposase* mRNA into one‐cell stage embryos [20] of the *Mitfa* ^*b692*^ */ednrb1* ^*b140*^ strain (ZIRC ID ZL1689). Three independent F1 lines showing robust and consistent pancreatic islet expression, and 50% transgene transmission, were maintained for further experiments. Previously published lines used in this study were: *ins:mCherry* [21], *pax6b:dsRed* [22], *neurod:memKate* [23].

All protocols used in this study were approved by the Austrian Bundesministerium für Wissenschaft und Forschung (GZ BMWF‐66.008/0009‐WF/II/3b/2014, GZ BMWFW‐66.008/0018‐WF/V/3b/2017 and GZ 2020‐0.282.289). Experiments were conducted in accordance with internationally accepted standards [24].

### Pharmacological treatments and feeding

Oligomycin (Sigma, Sigma‐Aldrich Gmbh, Vienna, Austria, 04876, stock solution: 5 mg·mL^−1^) was diluted to 3 μm in E3 media (5 mm NaCl, 0.17 mm KCl, 0.33 mm CaCl_2_, 0.33 mm MgSO_4_). H_2_O_2_ (30% with stabiliser, Roth, 8070.2, Carl Roth GmbH, Karlsruhe, Germany) was diluted to 750 μm in E3 media. Larvae were treated with H_2_O_2_ starting at 4 days post‐fertilisation (dpf), and imaged on day 6. The treatment solution was refreshed at 5 dpf. Larvae that were imaged between 7 and 8 dpf were fed with Zebrafeed (< 100 μm, Sparos, Olhão, Portugal) once per day starting at 5 dpf with a change of media each day.

### Microscopy

Larvae were anaesthetised in 0.003% Tricaine (Ethyl 3‐aminobenzoate, methanesulfonic acid salt, Acros, 118000500, Acros Organics, Geel, Belgium) and mounted in low melt agarose in 35 mm glass bottom dishes. To minimise jitter movement of the pancreas due to the close proximity of the beating heart, samples were overlaid with 0.2% Tricaine for the duration of image acquisition, as previously described [25]. Confocal fluorescence images were acquired with a Zeiss Axio Observer.Z1 equipped with a Yokogawa CSU‐X1 spinning disc using a 25× or 63× water‐immersion lens. For time lapse imaging, z‐stacks were scanned at intervals of 1–3 min. Longer or irregular intervals arise due to the need to reposition the sample following drift or gut movements. Bleaching of signal limited the duration of image series.

Imaging with a 63× objective had the limitation of phototoxicity and rapid bleaching, and few cells in the larval pancreas are situated sufficiently close to the surface for the limited imaging depth of the lens. We therefore confirmed the option of using a lower magnification objective (25× with 2.5× magnifying tube lens), which yielded accurate analysis of mitochondria, and images did not require deconvolution.

### Image processing and analysis

#### Registration and deconvolution

The image analysis workflow is summarised in Fig. S1. Initial image processing was performed using open‐source fiji software (https://fiji.sc/, [26]). Images were cropped to contain only the region of interest, then registered to correct for movement during z‐stack acquisition, using the plugins stackreg (for GFP only, http://bigwww.epfl.ch/thevenaz/stackreg/) or multistackreg (for 2‐colour stacks, [27]).

**Fig. 1:**
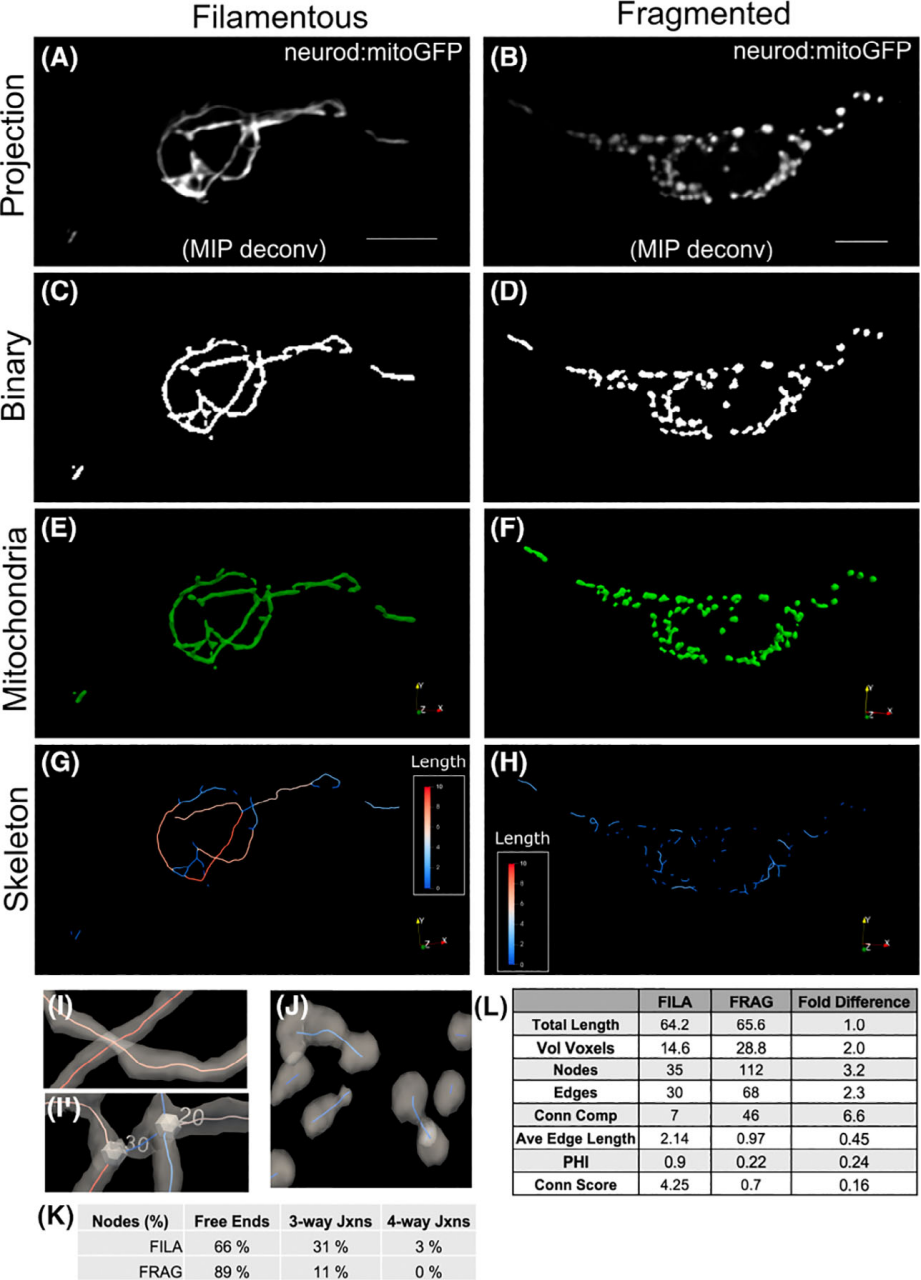
Quantitative analysis of mitoGFP‐labelled mitochondria in secondary islet cells using mitograph. Example cells showing contrasting mitochondria characteristics; filamentous (left) and fragmented (right). (A, B) Maximum intensity projection of *neurod:mitoGFP* image stacks following deconvolution. Scale bar, 5 μm. (C, D) Binary image following segmentation with mitograph v3.0. Visualisation of mitograph output using Paraview showing mitochondria (E, F) and skeleton (G, H). Skeleton segment lengths (μm) are as indicated by colour scale. (I, J) Close up views of the skeleton as in (G, H), combined with transparent mitochondria. Mitochondrial units that cross in different z‐planes are correctly separated (I) in contrast to mitochondria tubules joining at junctions, which are indicated as numbered nodes (I'). (J, K) The skeleton of fragmented mitochondria shows more free ends and fewer junctions compared to the filamentous network. (L) Comparison of quantitative characteristics (as explained in Materials and Methods) calculated for samples in (A, B). (Conn Comp, connected components; Conn Score, connectivity score; FILA, filamentous; FRAG, fragmented.).

Deconvolution (in Fig. 1) was performed using huygens professional (SVI, Scientific Volume Imaging B.V., Hilversum, The Netherlands, https://svi.nl/) using the CMLE algorithm, SNR (Signal‐to‐Noise Ratio) of 20, for 20 maximum iterations with a quality threshold of 0.01.

#### Mitochondria analysis

mitograph software (v3.0, https://github.com/vianamp/MitoGraph) performs 3D quantitation of image z‐stacks containing fluorescently labelled mitochondria [7, 28]. The software applies algorithms for stepwise enhancement of tubular elements, followed by image segmentation to identify mitochondrial objects (connected components). A further skeletonisation step permits network connectivity analysis. mitograph reports on total network length, nodes (endpoints and branch points), edges (mitochondrial tubules that extend between nodes), and the frequency and type of junctions. Average degree is the average number of edges per node. In addition, network connectedness is reported by PHI, which is the length of the largest connected component relative to the total mitochondrial length, and the connectivity score, which integrates multiple parameters into a single measure [7].

mitograph 3.0 was run through the terminal as described [7], with specification of image pixel size, z‐spacing and parameters as follows:

./mitograph3 ‐xy 0.104 ‐z 0.72 ‐adaptive 10 ‐path ~/Desktop/cells.

Samples with weaker signal had improved segmentation with altered ‘scales’ values:

./mitograph3 ‐xy 0.104 ‐z 0.72 ‐scales 1.5 2.0 6 ‐adaptive 10 ‐path ~/Desktop/cells.

R scripts to quantitate mitochondrial network properties [7] were used in rstudio (www.rstudio.com) as described. The generated text files were further processed using Excel. 3D surface models and skeletons were visualised using paraview (https://www.paraview.org/).

#### Cell segmentation and 3D measurements

For segmentation of cell boundaries, signal was smoothed using the Sigma Filter Plus (https://imagej.nih.gov/ij/plugins/sigma‐filter.html), followed by a median filter. Cell boundaries were defined semi‐automatically using the medical imaging interaction toolkit (mitk, https://www.mitk.org, [29]), by applying the functions Region Growing, LiveWire, and 2D interpolation in combination with manual adjustments using the Add and Subtract tools. Cell segmentations were saved as a ‘nii' file which was opened in fiji and smoothed using the ‘3D Binary Close Labels’ function of the 3D imagej suite [30]. Cell morphology quantitation was performed using the ‘Measure 3D’ function of the imagej 3D Manager Plugin in fiji [30], and the saved results were further processed using Excel. To separate mitochondria within adjacent cells, the segmentation masks were combined with the mitochondrial image stack using fiji (as described in the Supporting Information).

### Statistical analysis

Data were assembled in Excel and analysed using graphpad prism (vers 9.3, GraphPad Software, San Diego, CA, USA). In box plots the included dots represent individual data points, the centre line in the box indicates the median, the extent of the box represents interquartile ranges (25–75%), and the total range is shown in whiskers (0–100%). Two groups were compared using an unpaired t‐test, three groups were compared using one‐way ANOVA.

## Results

### *In vivo* imaging of endocrine cell mitochondria

In previous work, zebrafish mitochondria were imaged in the *Tg(EF1a:MLS‐EGFP)* transgenic line, which express mitochondria‐targeted (MLS, mitochondrial localisation signal) GFP from the *EF1a* promoter [31]. At 5 dpf, when the pancreas can be readily viewed in living animals, *EF1a:MLS‐EGFP* expression within the islet was weak compared to the surrounding exocrine pancreas (Fig. S2A,B). Although considered ‘ubiquitous’, the EF1a promoter is variably downregulated during development [32, 33] and the *EF1a:MLS‐EGFP* transgenic line appeared not optimal for studies of islet mitochondria. As we previously characterised the robust pancreatic islet expression driven from a 5 kb *neurod* promoter [23], we now generated a *neurod:mitoGFP* transgenic line to label mitochondria in pancreatic islet cells. In the newly established transgenic line, the *neurod* promoter directed robust expression of a mitochondrial targeted GFP within islet cells (Fig. S2C,D), providing a tool for imaging and analysing islet cell mitochondrial dynamics in zebrafish larvae *in vivo*.

The mitochondrial network within pancreatic islet cells, as labelled by *neurod:mitoGFP*, formed a highly complex network (Fig. S2E), showing long and short filaments, as well as loops and more densely packed clumps. This is similar to previously reported mitochondrial complexity in human beta cells in culture [34]. In combination with *ins:mCherry* expression, mitochondria within beta cells could be observed (Fig. S2E).

### Quantification of mitochondria in single islet cells

While the mitochondrial network within the islet was clearly visible, previously developed software applications have mainly been utilised for mitochondrial analysis within isolated, adherent cells growing in culture, and cannot readily be applied to such a complex structure. To develop quantitative methods applicable for islet cell mitochondria *in vivo*, we took advantage of the islet developmental process, during which progenitors differentiate as single cells along the pancreatic duct and progressively aggregate to form the clustered endocrine islet [35, 36]. In zebrafish, these cells begin to appear at 4–5 dpf (Fig. S2D), when the larval pancreas is superficially located and highly amenable to *in vivo* imaging.

We first set out to establish a pipeline for analysis of mitochondrial morphology in isolated cells, a more tractable task as compared to unravelling the mitochondrial network of the whole islet. For this we analysed representative cells from 7 dpf *neurod:mitoGFP* larvae with mitochondrial network characteristics readily distinguished by visual inspection, namely a filamentous as compared to a fragmented network, likely induced here by cell stress due to phototoxicity [7]. Cropped z‐stack images containing single cells were processed for mitochondrial morphology using the validated software mitograph (v3.0), which provides 3‐dimensional (3D) quantitation and has been applied in cultured mammalian cells [7, 28, 37]. Comparison of the binary images against z‐projections of the original images demonstrated a good discrimination between fluorescently labelled mitochondria and background (Fig. 1A vs. C, B vs. D). mitograph software accurately identified mitochondrial elements (Fig. 1E,F) and executed skeletonisation of the network (Fig. 1G,H). Close‐up visualisation confirmed that mitochondria crossing at different z‐levels were detected as distinct tubules, in contrast to multiple tubules meeting at a junctional node (Fig. 1I,I′). The fragmented network appeared in 3D as spherical and short elements (Fig. 1J). As compared to the fragmented network, the highly interconnected filamentous network had fewer free ends, and more nodes where three or four tubules meet (three‐ and four‐way junctions, Fig. 1K).

mitograph generates a count of discrete mitochondrial objects (connected components), and provides analysis of the mitochondrial skeleton to yield number and connectivity of nodes (ends and branch points) and edges, as well as average edge length. Consistent with a visual assessment, the filamentous mitochondrial network had fewer connected components, fewer nodes, and longer edges compared to the fragmented network (Fig. 1L). The PHI value close to one indicated that the majority of tubules were joined in a single complex element. Total mitochondrial length was comparable between the two cells. The mitochondrial connectivity score (Conn Score), which combines the network parameters into single value, was six‐fold higher in the filamentous network (Conn Score = 4.25), as compared to the fragmented network (Conn Score = 0.7).

Mitochondrial network fragmentation in response to inhibition of mitochondrial function has been demonstrated in cultured cells [38]. To examine the response to mitochondrial perturbation of islet cells *in vivo*, 7 dpf larvae were treated with the ATP synthase inhibitor oligomycin. Consistent with previous work [39], treatment with 3 μm for 1 h reduced motor responses and heartbeat. Based on visual assessment and the mitograph connectivity score, a minor population of islet cells in oligomycin‐treated samples had mitochondria which resembled controls (5/16, ‘mild’, Fig. S3A,B,D,E), while in the majority of islet cells in treated samples, the network was fragmented and had a connectivity score < 3.0 (11/16, ‘fragmented’, Fig. S3C,F,G). Further parameters were also consistent with fragmentation, including increased connected components (Fig. S2H). The disrupted network connectivity was apparent in the increase of nodes that occurred as free ends (Fig. S3I), and the decrease of three‐way junctions as compared to controls (Fig. S3J). The cells with a mild phenotype resembled controls in the connectivity score (Fig. S3G), number of connected components (Fig. S3H), and showed a trend towards the fragmented cell phenotype in free ends and three‐way junctions (Fig. S3I,J). Such mildly affected cells likely arise due to variability in the response of individual cells, as we detected different responses of mitochondria in cells within the same sample.

### Islet cell mitochondrial responses to oxidative stress

With this demonstrated capability to distinguish and quantitate mitochondrial phenotypes within the intact organism, we next examined mitochondrial responses to pathophysiological manipulation. Beta cells are suggested to be susceptible to oxidative stress due to relatively low antioxidant capacity [40], and excessive reactive oxygen species (ROS) resulting from glucotoxicity may contribute to diabetes pathogenesis. To determine the response of islet cell mitochondria to oxidative stress, larvae were treated with H_2_O_2_ for 48 h and imaged at 6 dpf. In combination with *neurod:mitoGFP*, we included the *pax6b:dsRed* transgene, and used the red cytoplasmic fluorescence in endocrine cells to segment and separate instances of overlapping cells (Figs S1 and S4A–F).

**Fig. 2:**
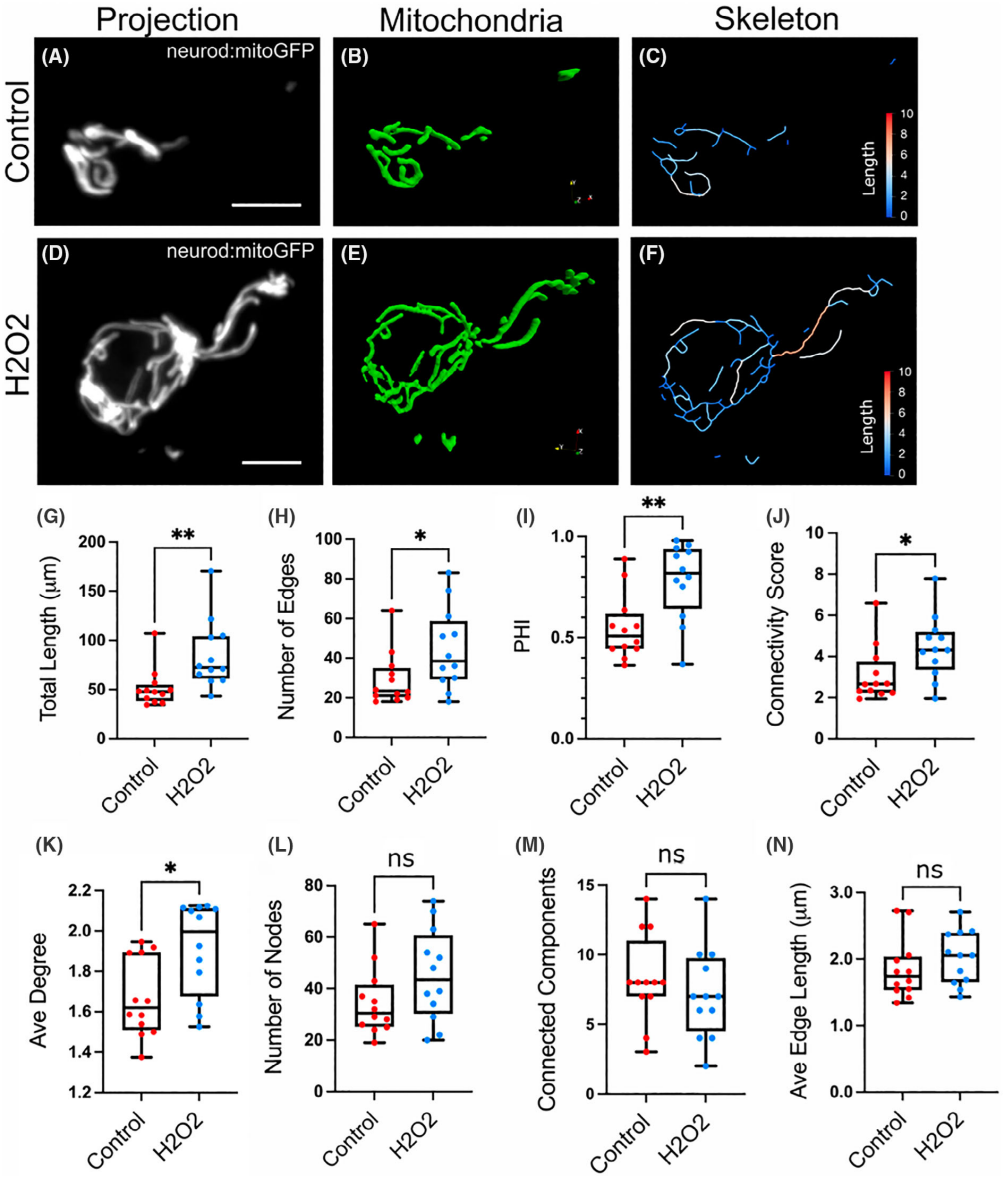
Treatment with H_2_O_2_ causes mitochondrial expansion. Representative cells from *neuro:mitoGFP* larvae at 6 dpf, control (A–C) and H_2_O_2_‐treated (D–F). (A, D) Maximum intensity projection of *neurod:mitoGFP* (grey). Scale bar, 5 μm. Paraview 3D representation of the mitochondrial objects (B, E) and the skeletonised network (C, F) following analysis with mitograph. Skeleton segment lengths (μm) are as indicated by colour scale. (Details of analysis pipeline are in the Methods and Fig. S1.) (G–N) Quantification of mitochondrial parameters in islet cells of control versus H_2_O_2_‐treated larvae. Mitochondrial length (G), number of edges (H), PHI (I) Connectivity Score (J) and Average Degree (K) increased with treatment, number of nodes (L), connected components (M) and average edge length (N) did not significantly change with H_2_O_2_ treatment. *P < 0.05, **P < 0.01, ns not significant. (Results combined from three independent experiments, control, *n* = 12; H_2_O_2_ treated, *n* = 12).

In H_2_O_2_‐treated cells, the mitochondrial network appeared to be expanded as compared to controls (Fig. 2A–F). Quantitative parameters supported this observation, with H_2_O_2_‐treated cells showing an increase in total length of the network and more edges (Fig. 2G,H). PHI and the connectivity score were increased (Fig. 2I,J), consistent with an increase in the average degree (number of edges per node, Fig. 2K). The number of nodes, connected components and average edge length were not significantly different between control and H_2_O_2_‐treated cells (Fig. 2L–N). Thus, the increase in mitochondrial length in response to H_2_O_2_ coincided with an increase of edges, to generate an extended and more interconnected network.

### Mitochondria analysis in multicellular clusters

**Fig. 3:**
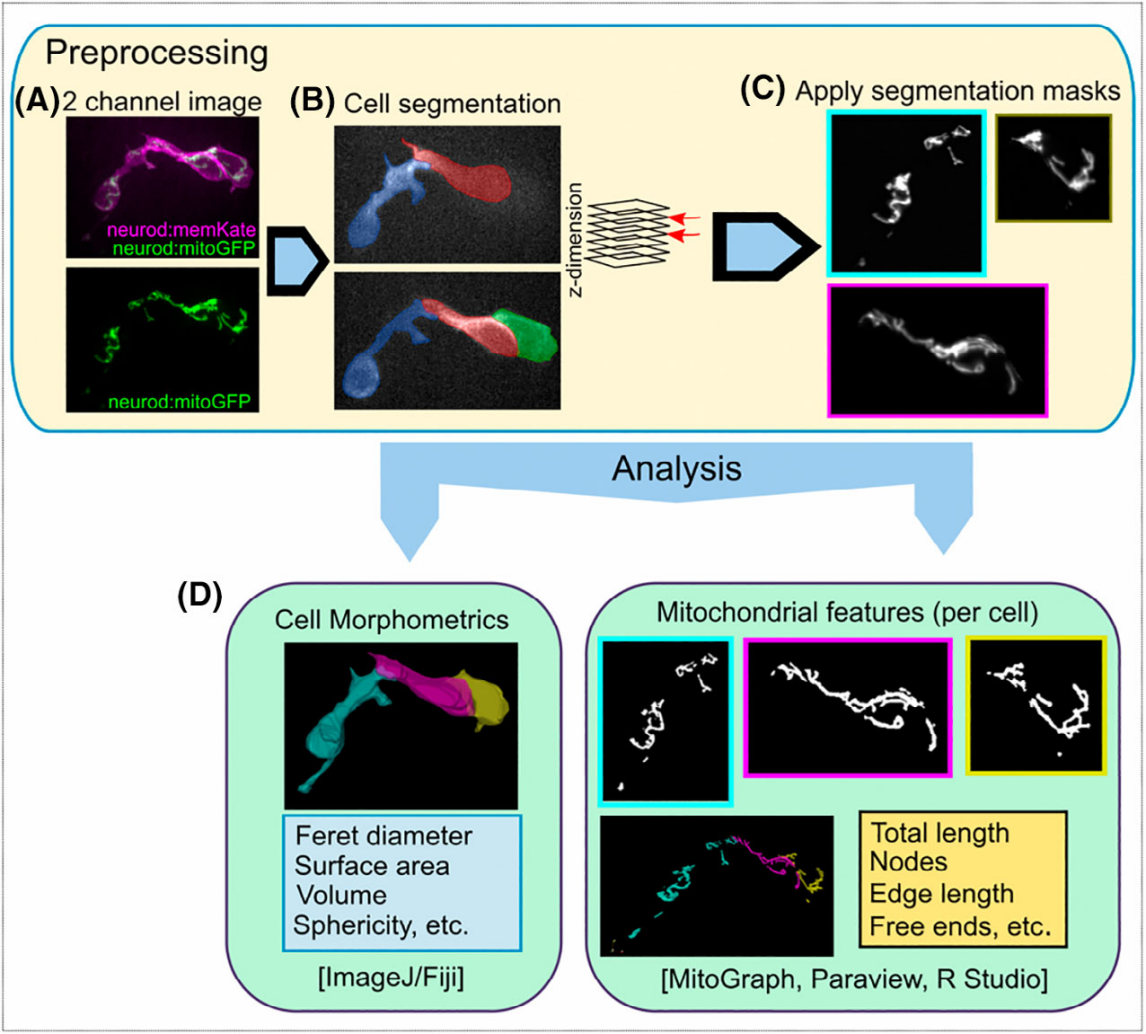
Segmentation of mitochondria in clustering cells. (A) Expression of *neurod:memKate* in addition to *neurod:mitoGFP* facilitates segmentation of mitochondria in adjoining cells. Closely opposed mitochondria cannot be assigned to specific cells based on *neurod:mitoGFP* expression alone (A, bottom). (B) Cell segmentation, shown in two different z‐planes, based on the membrane‐localised memKate signal. (C) By using cell segmentation masks, mitochondria from each cell can be separated. (D) Quantitative analysis of single cell morphology (left) and the corresponding mitochondrial network (right) using open‐source software.

One limitation described for mitograph is the necessity of having isolated single cells [7]. To study mitochondria during developmental processes, the mitochondria of cells in contact with each other and even in clusters must also be rendered amenable to analysis. In *neurod:mitoGFP;pax6b:dsRed* double transgenics, *pax6b:dsRed* provides information for cell segmentation but transgene signal accumulates centrally, and edges at contact points between adjacent cells are not well defined (Fig. S4B,G). This is problematic for analysing cell clusters, as the peripherally dispersed islet cell mitochondria network cannot be easily assigned to the corresponding cell. Therefore, we used the membrane labelling of the *neurod:memKate* transgenic line [23] to distinguish boundaries of cells in clusters and to generate masks for cell segmentation (Fig. 3A,B). By combining masks with the original image, z‐stacks containing the mitochondria of individual cells were generated (Figs 3C and S5A,B). Analysis of these stacks with mitograph frequently did not yield satisfactory results, as spurious signal was produced (Fig. S5E). We reasoned that thresholding and segmentation performed poorly due to the sharp pixel intensity difference between the outside and inside of the cell due to our masking process. To correct this, based on the previously published method [7], we included the addition of Gaussian‐type noise external to the cell mask boundary prior to processing with mitograph. This simulated irregular background signal intensities (Fig. S5C,D), improving the analysis results of mitograph (Fig. S5E), to distinguish mitochondria in clustered cells (Fig. 3D).

### Mitochondrial network elaboration during islet formation

**Fig. 4:**
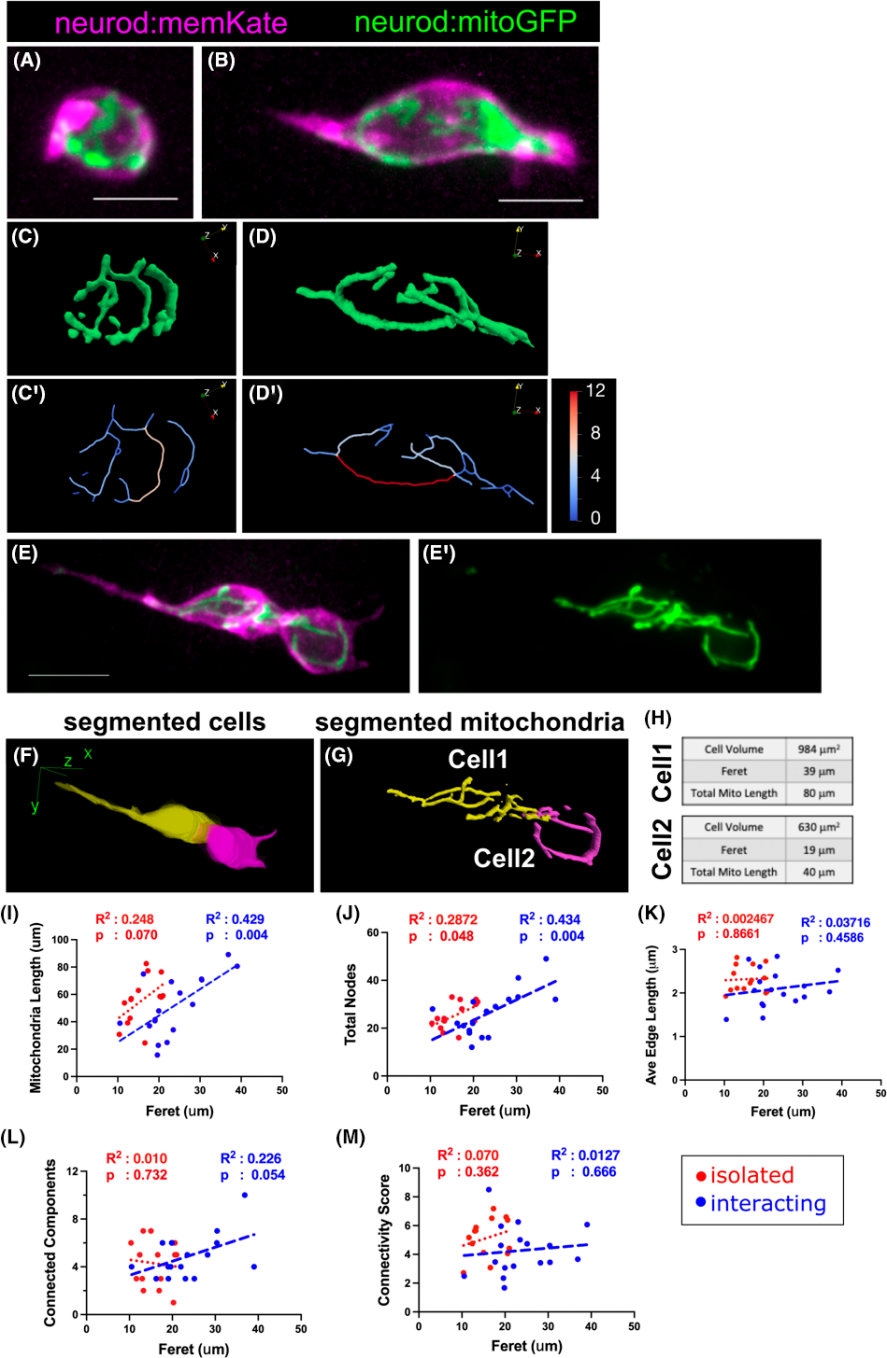
Mitochondrial morphologies of clustering islet cells. (A, B) Maximum intensity projection of image stack of isolated islet cells from *neurod:mitoGFP;neurod:memKate* transgenics at 7 dpf. Mitochondrial network (C, D) and skeleton (C′, D′) as analysed by mitograph. Skeleton segment lengths (μm) indicated by colour scale. (E–H) Mitochondrial analysis in adjoining cells. (E, E′) Confocal stack projection showing mitochondria (green) with cell boundaries (magenta) (E) and mitochondria alone (E′). (F) Segmentation of adjacent cells, and their corresponding mitochondria (G) based on memKate expression. (H) Calculated values for cell volume, Feret diameter and total mitochondrial length in cells shown in (F, G). (I–M) Linear regression analyses comparing mitochondrial parameters to Feret diameter in isolated (red, as in A, B) versus interacting (blue, as in E) islet cells at 7–8 dpf. Regression lines are shown, with *R* ^2^ and p values as indicated. Results combined from three independent experiments, isolated cells *n* = 14; interacting cells *n* = 15. (A, B, E; Scale bar, 10 μm).

Mitochondrial topology adapts to cell state and metabolic needs, with a progression from fragmented to elongated forms characterising the transition from progenitor to a more differentiated state [1]. Previous studies showed that the endocrine cells are very dynamic during islet morphogenesis, as progenitors differentiate and coalesce into clusters [23, 35, 36], but mitochondria have not been examined during this process. To investigate mitochondria morphology and distribution in relation to islet assembly, we analysed larvae expressing *neurod:memKate*, in addition to *neurod:mitoGFP*. In compact cells, filamentous mitochondria were distributed at the periphery of the cell (Fig. 4A,C). In cells with more expanded morphologies, mitochondria extended into cellular protrusions (Fig. 4B,D). The cell membrane labelling by *neurod:memKate* further enabled the separation of cells in direct contact and within small clusters, and to distinguish their mitochondrial components (Fig. 4E–G). Mitochondria in adjacent cells showed close apposition at regions of cell–cell contact (Fig. 4G). Clustering cells showed a range of morphologies from compact to extended with long protrusions (Fig. 4E,F), with corresponding variability in mitochondria length and cell volume (Fig. 4H). Feret diameter, an indicator of an extended cell morphology, similarly associated with increased total mitochondrial length (Fig. 4H).

To further explore the connection between mitochondrial network dynamics and cell morphology during islet formation, we analysed the relationship between mitochondrial parameters and Feret diameter in isolated compared to interacting and clustering cells. An increasing Feret diameter correlated with a trend towards increasing mitochondria network length in isolated cells (*P* = 0.070, *n* = 14), and this relationship was highly significant (*P* = 0.004, *n* = 17) in interacting cells (Fig. 4I). The increase in total mitochondrial length was achieved through addition of new segments, as the number of nodes increased, while average edge length remained basically unchanged (Fig. 4J,K). The number of connected components and the connectivity score did not vary in relation to Feret diameter (Fig. 4L,M). Overall, this indicates mitochondrial expansion was coupled to cell maturation and to the increase of cell motility characteristic of endocrine cells during islet formation. Moreover, a highly interconnected and filamentous mitochondrial network persisted during the process of cluster formation.

### Directed mitochondrial redistribution to regions of dynamic motility

**Fig. 5:**
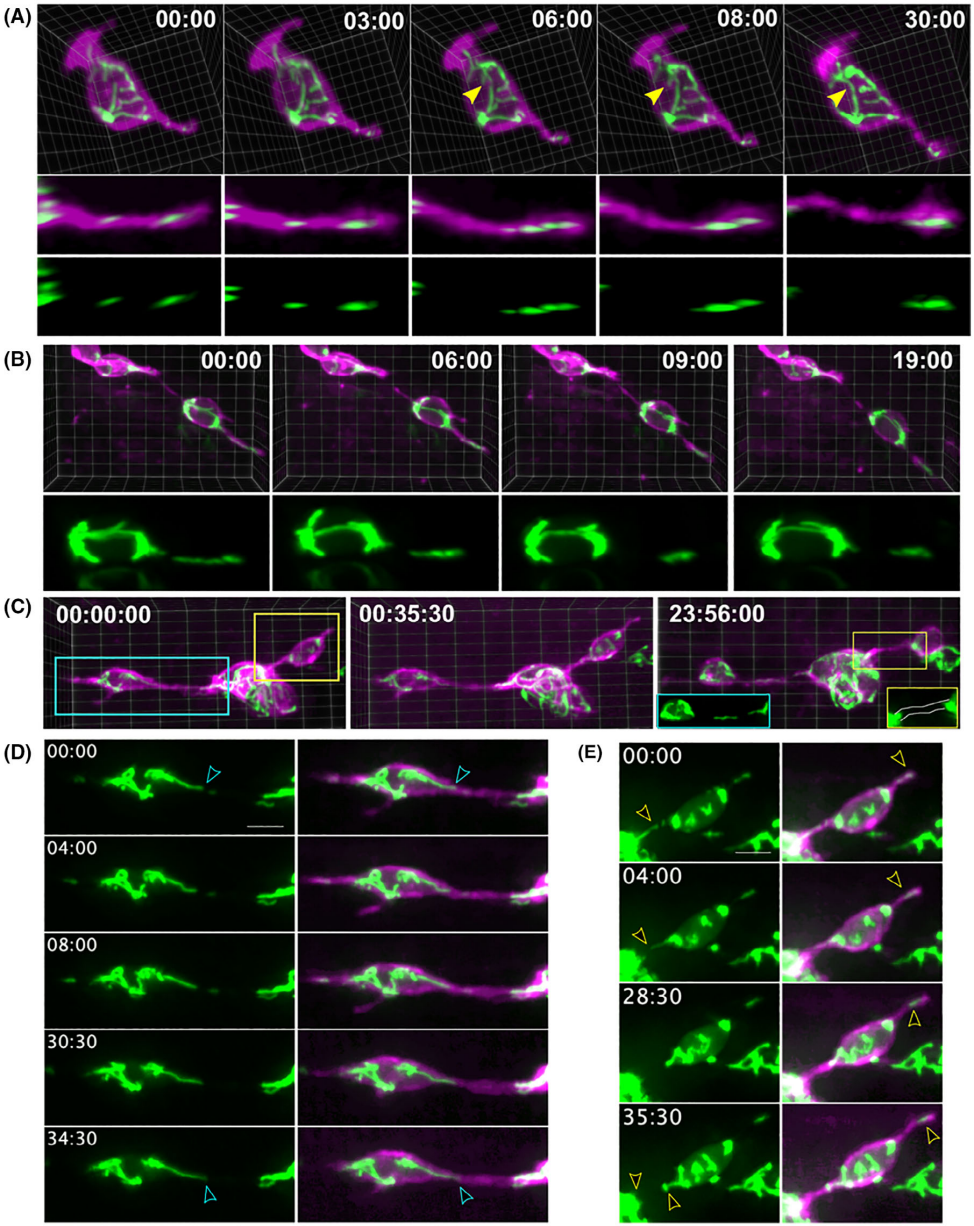
Mitochondrial localisation in relation to cell motility. (A, B) Mitochondria within protrusions move as discrete units (A) or elongated filaments (B). Distally directed mitochondrial displacement over time shown in 3D visualisations of the full cell (A, top), and a rotated view to show the full protrusion length (A, middle), and the mitoGFP signal alone (A, bottom). 3D visualisation of two nearby cells (B) with a filamentous mitochondria shifting distally within a protrusion. (C) 3D view of mitochondria dynamics within clustering cells. When visualised the next day (C, right), the filamentous protrusion has detached from the cell body network (blue inset) and the second cell–cell connection is devoid of mitochondria (yellow inset). (D, E) z‐stack projections highlighting the regions of (A) within the blue box (D) and yellow box (E). (A, B, D, E) Times are as indicated: min : s. (C) Times are as indicated: h : min : s. (D, E) Scale bar, 5 μm.

Although the above studies suggest that the overall mitochondrial network characteristics remained relatively stable during islet assembly, there was generation of new mitochondria in extended cells. We know from previous studies that these cells are highly motile [23, 35], and in still images we observed a striking distribution of discrete mitochondrial units at proximal to distal locations in long cellular protrusions (Fig. S6A,B). We therefore hypothesised that mitochondria are actively translocated into protrusions to provide energy for cell motility, as has been described for migrating cell types and neurons [2]. To start to decipher this redistribution process, we performed time lapse imaging. In emerging islet cells of *neurod:mitoGFP;neurod:memKate* double transgenics at 7–8 dpf, mitochondria movements were readily observed within the cell body of both isolated and clustering cells. Types of movements observed included fission and fusion, while filaments displayed extension, retraction and branching, as well as translocations (Figs 5A and S7A,B, and Videos S1 and S2).

Similar to what is seen in axons [14], detached mitochondrial units translocated from proximal to more distal locations within a protrusion, and localised near protrusion tips (Figs 5A and S7A). We also observed an alternative mode where mitochondrial filaments extended within a protrusion (Fig. 5B). Over the imaging period, the gap of separation to the cell body network widened and the mitochondria shifted distally (Fig. 5B). Within loosely associated cell clusters, mitochondrial movements could be detected in protrusions and cell–cell connections (Fig. 5C–E). Extension of a protrusion was accompanied by distally directed mitochondrial movements (Fig. 5E). Cell–cell bridges persisted over timespans up to 24 h, as demonstrated in an example in which a previously imaged cell cluster was observed on the subsequent day (Fig. 5C, right). At the later timepoint, a filamentous mitochondria was localised to the centre of a connecting protrusion where there had previously been an extension from the cell body network (Fig. 5C, right, D). The other cell–cell connection that initially contained mitochondria was devoid of mitochondria on the next day (Fig. 5C, right, E). We further determined that mitochondrial motility persisted within clustered cells, as we observed extension and retraction of peripheral filaments, and shape changes of more centrally located mitochondrial units (Fig. S8, Video S3).

While half of the protrusions showed mitochondrial movements, in 50% of cases (6/12), mitochondria within long protrusions varied in shape but maintained a stable position relative to the long axis over observation times of up to 1 h (Fig. S7B). Overall, this suggests that mitochondria are not required for maintenance of stable protrusions, and that multiple mechanisms contribute to mitochondrial distribution within protrusions, implicating cellular machinery acting to promote translocation as well as serving to anchor mitochondria in place.

## Discussion

Here, we have examined mitochondria network properties and behaviours *in vivo*, within the context of the developing pancreatic islet. For this we generated new transgenic lines and developed a pipeline for quantitative assessment of mitochondria morphology, based on open‐source software applications. Notably, we expanded the applicability of mitograph software to enable analysis of mitochondria from cells in close proximity and in clusters. The pancreatic islet showed a highly complex and dense network of filamentous mitochondria, and we focused our analysis on the more tractable single cells and small clusters that represent precursors for newly forming islets.

In response to exogenous H_2_O_2_, there was an increase in mitochondrial length and connectivity, which could be due to expansion or mitochondrial biogenesis, which serves to increase the capacity for quenching oxidative stress [41]. While many stressors shift the balance between fission and fusion, mitochondria can also transition to elongated morphologies through fusion‐independent processes [42]. Our studies did not distinguish between these alternatives. One limitation of this work is a potential confounding influence of genetic background on metabolic responses [43], including mitochondrial dynamics. This could be minimised by using inbred strains [44], but this is a complex task when there is a need to combine multiple pre‐existing lines.

Studies of mitochondria in native tissue are infrequent, and have revealed differences in mitochondrial structure between intact tissues and cultured cells. For example, mitochondria of cultured cardiac myocytes show active movements, which contrasts with the predominantly motionless mitochondria seen within intact cardiac tissue [45]. Mitochondria in cultured endothelial cells are filamentous and networked, while more fragmented mitochondria were seen in fixed samples of intact aorta [46], and in mouse cerebral vasculature using *in vivo* microscopy [47]. In human pancreatic cells in culture, mitochondria show highly interconnected, filamentous networks, and display complex movement dynamics, including fission, fusion and translocations [34]. Our work, which used a fluorescent protein targeted to the inner mitochondrial membrane, importantly confirms that previously reported *in vitro* mitochondrial dynamics are also a characteristic of islet cells in their native tissue environment. Further insights into mechanisms controlling beta cell mitochondria behaviours will emerge by adapting new probes and super‐resolution imaging technologies to organ culture and *in vivo* models. Probes tethered to the outer mitochondrial membrane are advantageous for revealing mitochondrial dynamics at high resolution [48] while novel, superresolution‐compatible dyes labelling the inner mitochondrial membrane delineate sub‐mitochondrial structures, including cristae [49].

Active mitochondria redistribution and remodelling serve to localise mitochondria to subcellular regions with increased energy needs. In coalescing islet cells, we saw mitochondrial expansion correlating with an extended cellular morphology, consistent with a need to supply mitochondrial units which provide energy to cytoplasmic protrusions that participate in the islet assembly process. Previous studies have observed that mitochondria dynamically infiltrate protrusive structures involved in cell migration, but mechanisms driving this redistribution vary with cell type [2]. Neutrophil mitochondria remain highly networked during cell migration, while mitochondrial fragmentation promotes cell motility in T cells and during cancer cell invasion [50, 51]. Long distance transport of mitochondria has been defined for neuronal processes, but has not been well described in other cell types. Here we observed mitochondrial distribution at proximal to distal locations in cellular extensions. The morphology of mitochondria within protrusions ranged from small fragments to extended filaments, while network quantitation showed that the average edge length and network connectivity remained consistent over a range of cell shapes. This argues against a predominant mechanism of widespread network fragmentation supplying mitochondrial units to protrusions. However, uninterrupted filaments extending into long protrusions were only infrequently observed.

In this work, technical limitations of photo‐bleaching and phototoxicity limited our ability to follow the full evolution of mitochondrial behaviours within protrusions. Thus, the mechanism of mitochondrial entry into protrusions requires further clarification, which can be aided by using photoactivatable transgenes [15] to enable long‐term tracing. Future experiments, combining genetic and pharmacologic reagents with live imaging, can define the signals and molecular machinery that drive mitochondrial network modulation and relocalisation.

In cultured cells, mitochondrial analysis following dye labelling is confounded by uneven dye uptake and a lack of cell‐type specificity. In our system, we have cell type specificity but transgene expression initiates with cell differentiation, and signal accumulates over time. As detection depends on signal strength as well as cell position within the tissue, we could not avoid some variability in image quality, which necessitates user intervention and tuning in our processing pipeline. This may not be the case for other applications of our approach, and preprocessing and cell segmentation steps can be adapted and automated according to the individual experimental situation. Incorporation of emerging methods to remove noise, equalise signal across samples, and correct for *z*‐axis undersampling [52] can yield more uniform signal quality and increase consistency of processing parameters.

Accumulating evidence suggests that beta cell mitochondrial dysfunction may be both a causal and an exacerbating factor in the dysregulated glucose homeostasis of diabetes [53], but these interrelationships remain poorly defined. Expression of mitochondria morphology regulators is altered in rodent diabetes models, and manipulation of mitochondrial dynamics impacts susceptibility of beta cells to apoptosis [54, 55], which has relevance for development of diabetes therapeutics. Furthermore, mitochondrial features that evolve in parallel to islet maturation may provide informative measures for monitoring *in vitro* differentiation to generate functionally mature replacement islet cells for diabetes therapies. Overall, our newly developed methods can be applied for *in vivo* analysis of islet cell mitochondria under normal and disease conditions, and can be adapted to delineate mitochondrial dynamics in other organ systems during development, homeostasis and disease.

## Author contributions

JF developed tools, performed experiments and edited the manuscript. DM provided resources and supervision, and edited the manuscript. RAK designed the study, performed experiments, analysed data, acquired funding, performed supervision, and drafted and revised the manuscript.

## Supporting information


**Fig. S1.** Schematic of analysis pipeline for single and clustered cells.
**Fig. S2.** Mitochondrial transgene expression in pancreas and islet.
**Fig. S3.** Inhibition of mitochondrial function alters mitochondrial morphology.
**Fig. S4.** Segmentation of overlapping cells.
**Fig. S5.** Analysis of mitochondria from clustered cells requires addition of noise.
**Fig. S6.** Mitochondria distribution in islet cell protrusions.
**Fig. S7.** Dynamic mitochondrial behavior captured in time lapse images.
**Fig. S8.** Mitochondrial motility in clustered cells.

**Video S1.** Time lapse movie with image capture at the indicated times. 3D visualization of isolated islet cell expressing *neurod:mito‐GFP* (mitochondria, green) and *neurod:memKate* (cell membranes, magenta) Related to Fig. 5A.

**Video S2.** Time lapse movie with image capture at the indicated times. 3D visualization of isolated islet cell expressing *neurod:mito‐GFP* (mitochondria, green) and *neurod:memKate* (cell membranes, magenta). Related to Fig. S7B.

**Video S3.** Time lapse movie with image capture at the indicated times. 3D visualization of a small endocrine cell cluster showing (left) labeled mitochondria (green, *neurod:mito‐GFP*) and cell membranes (magenta, *neurod:memKate*), GFP channel alone for clarity (right). Related to Fig. S8.

## Data Availability

The data supporting the findings of this study are available within the article and the supporting information. Additional data that support the findings of this study are available from the corresponding author upon reasonable request.
