# Supplementary material for: Mitochondrial network expansion and dynamic redistribution during islet morphogenesis in zebrafish larvae
Source: FEBS Lett. 2022 Oct 19;597(2):262–75. doi: 10.1002/1873-3468.14508 (PMC10092693; doi:10.1002/1873-3468.14508)
Supplement: Supplementary file 1 — Fig. S1. Schematic of analysis pipeline for single and clustered cells. Fig. S2. Mitochondrial transgene expression in pancreas and islet. Fig. S3. Inhibition of mitochondrial function alters mitochondrial morphology. Fig. S4. Segmentation of overlapping cells. Fig. S5. Analysis of mitochondria from clustered cells requires addition of noise. Fig. S6. Mitochondria distribution in islet cell protrusions. Fig. S7. Dynamic mitochondrial behavior captured in time lapse images. Fig. S8. Mitochondrial motility in clustered cells. [file FEB2-597-262-s002.pdf]

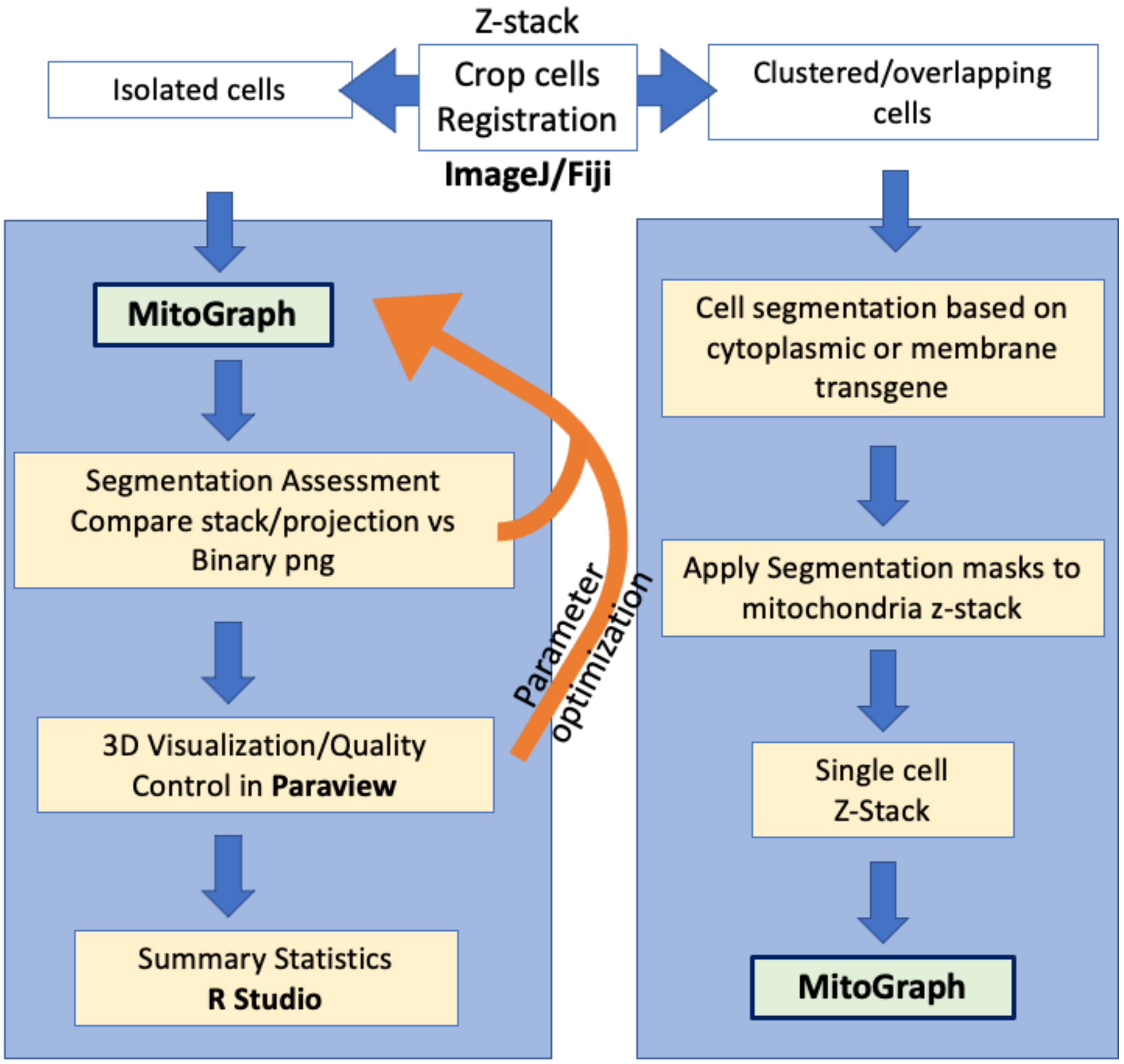

Supplementary Figure 1. Schematic of analysis pipeline for single and clustered cells.

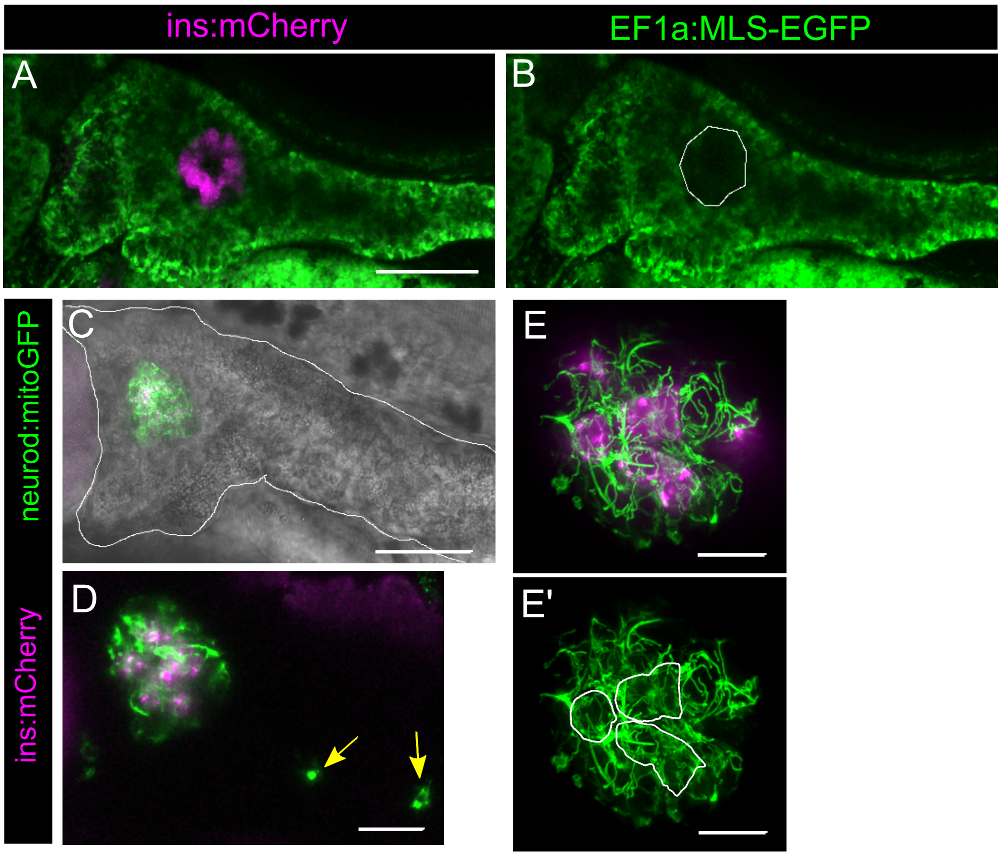

Supplementary Figure 2. Mitochondrial transgene expression in pancreas and islet.
(A, B) Mitochondria-localized MLS-EGFP expression in the pancreas (green) and Ins-expressing beta cells (magenta) at 5 dpf. **(A)** Maximum intensity projection of confocal image stack. Scale bar, 50μm. **(B)** MLS-EGFP expression shown alone for clarity, expression is weak within the islet (white outline). **(C)** Single z-plane image of anterior pancreas in *Tg(neurod:mitoGFP; ins:mCherry)* larva at 5 dpf. Fluorescent image (mitochondria, green; beta cells, magenta) merged with brightfield. Pancreas is outlined in white. Scale bar, 50 μm. **(D)** Confocal z-stack projection of *neurod:mitoGFP* (green) and *ins:mCherry* (magenta) expression in the pancreatic islet at 5 dpf. Yellow arrows indicate newly differentiating secondary islet cells. Scale bar, 20 μm. **(E)** Maximum intensity projection of the central islet of a sample as in **(D,** mitochondria, green; beta cells, magenta). **(E')** GFP channel alone. White outline indicates ins:mCherry+ beta cells. Scale bar, 10 μm.

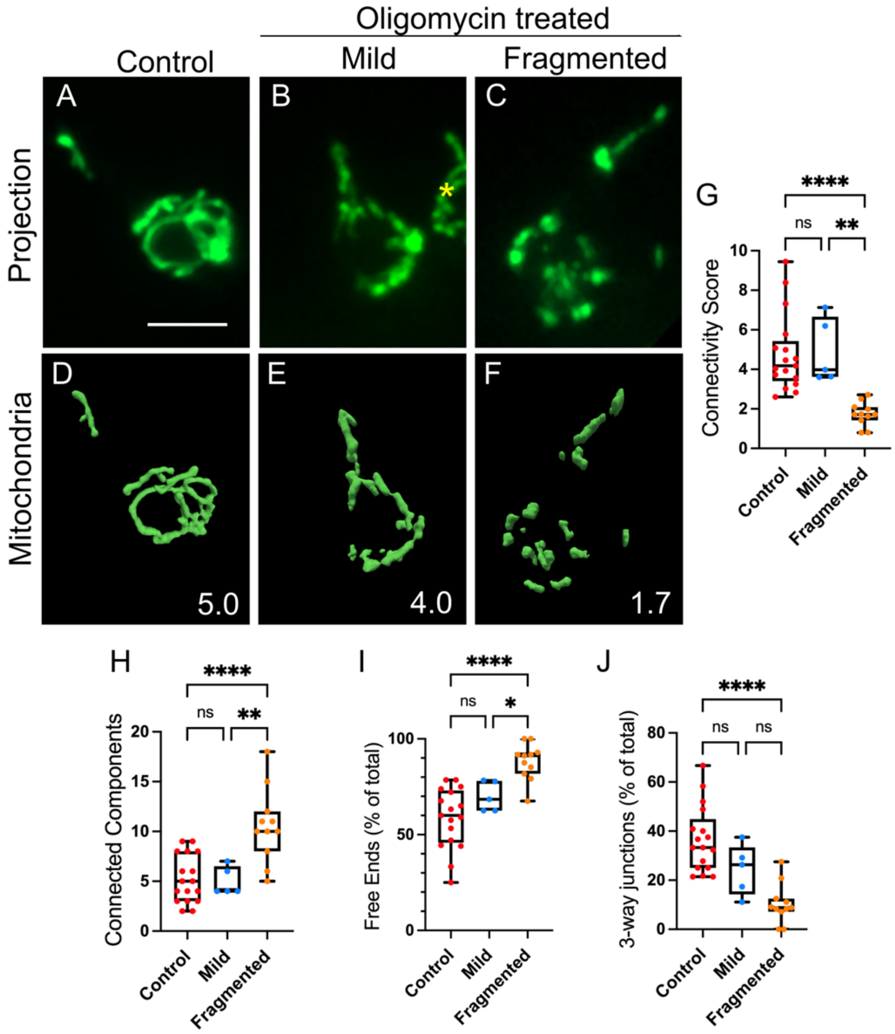

Supplementary Figure 3. Inhibition of mitochondrial function alters mitochondrial morphology.

Maximum intensity projections (**A-C**) and 3D mitochondria representations (**D-F**) of controls (**A, D**) and larvae treated with 3 µm oligomycin for one hour (**B-C, E-F**). Number in (**D-F**) indicates connectivity score. Scale bar, 5 µm. (**G-J**) Quantification of mitochondrial parameters. Cells with fragmented mitochondria (n=11/16) had a MitoGraph connectivity score < 3.0 (**G**), elevated connected components (**H**), increased free ends (**I**) and decreased 3-way junctions (**J**). $^*p < 0.05$, $^{**}p < 0.01$ $^{****}p < 0.0001$, ns, not significant. Control, n=17; Oligomycin treated, n=16. Yellow asterisk (in **B**) indicates mitochondria from an adjacent cell that were not included in the analysis. (Results combined from 2 independent experiments.)

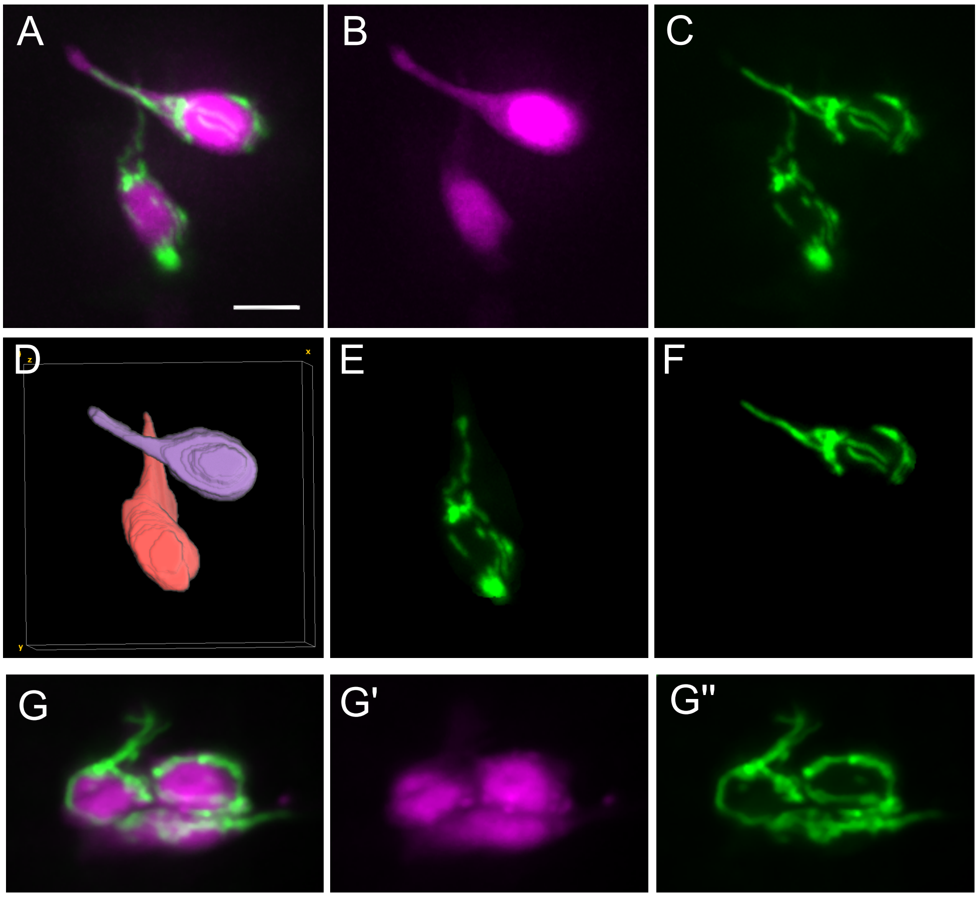

Supplementary Figure 4. Segmentation of overlapping cells.
(A-C) Mitochondria of overlapping cells expressing *neurod:mitoGFP* (green) and *pax6b:dsRed* (magenta) cannot be separated by drawing a region of interest on an image projection. Scale bar, 5 μm. (**D**) 3D view showing cell masks generated in MITK, based on cytoplasmic *pax6b:dsRed* transgene expression. Following segmentation based on these masks, the cells' overlapping mitochondria can be separated **(E, F)**. **(G)** *pax6b:dsRed* expression is weak in the cell periphery, which hinders the separation of mitochondria in clustered cells. Fluorescent transgene images (**A-C**, **E-G**) are maximum intensity projections of image stacks.

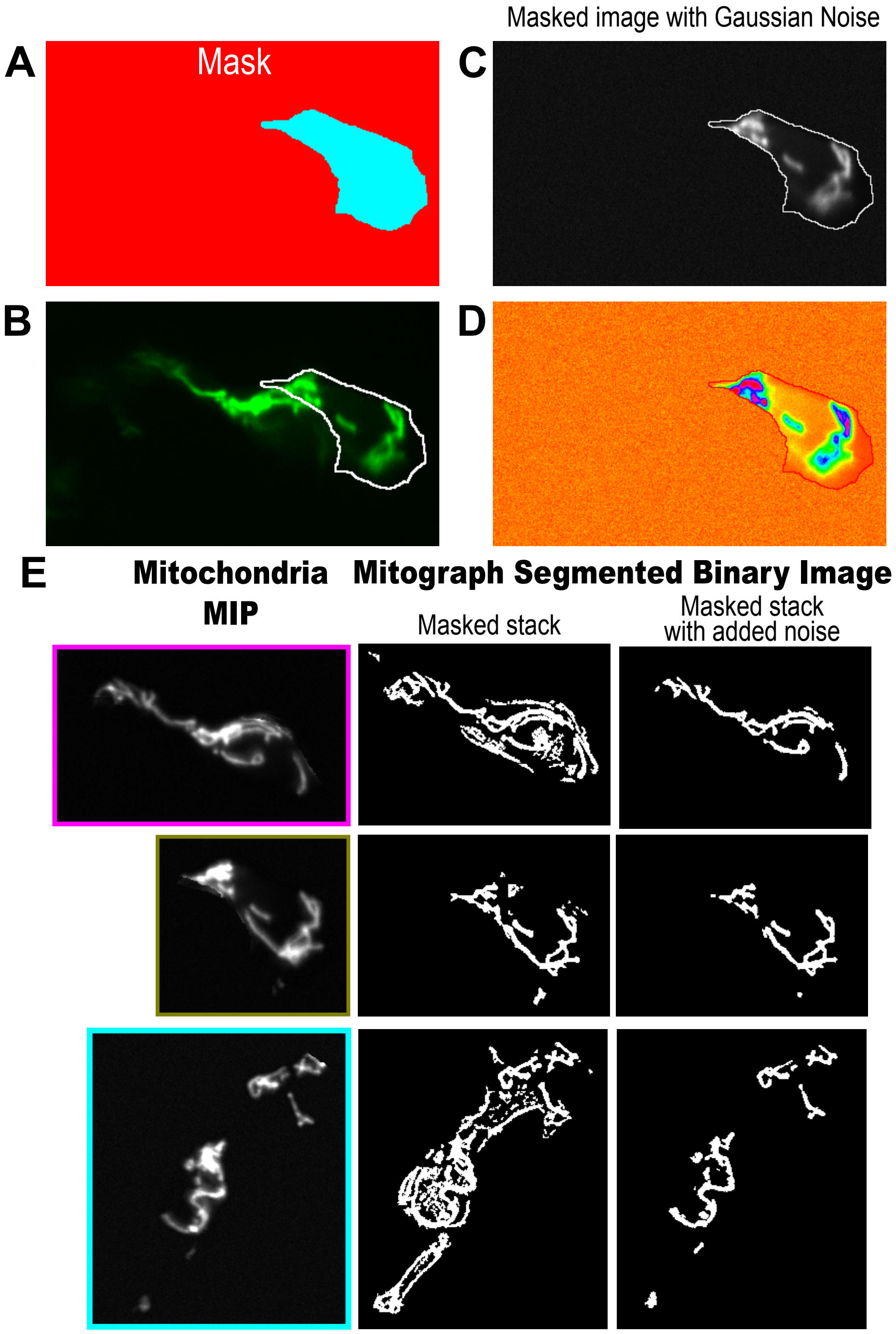

Supplementary Figure 5. Analysis of mitochondria from clustered cells requires addition of noise.

(A-B) Mitochondria in adjacent cells can be isolated based on a slice-by-slice application of the cell segmentation mask **(A)** to the original image **(B).** Signal within the white outline will be maintained, and signal external to this region will be removed. (Cells are shown in Fig. 3A.) **(C-D)** Adding a noise background to the segmented image improves the accuracy of subsequent mitochondrial analysis. **(E)** Mitograph analysis of mitochondria signal in image stack shown as a z-projection (left). The binary image generated from the segmented image stack (center) contains spurious signals not found in the original image. Addition of a noise background to the image stack (as in **D**) reduces the erroneous signals and yields correctly identified mitochondria. **A** and **D** are shown with the Lookup Table 'Spectrum' to highlight the addition of noise in **D**.

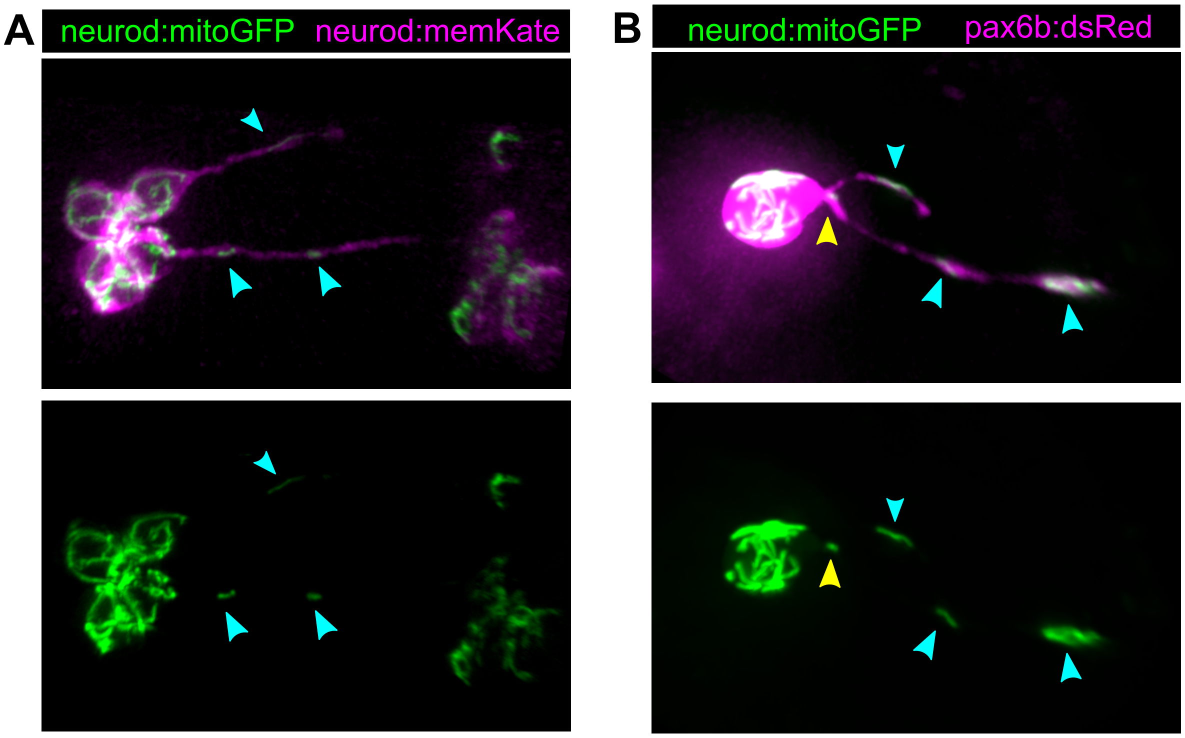

Supplementary Figure 6. Mitochondria distribution in islet cell protrusions.
3D visualization of confocal image stacks showing endocrine cell mitochondria (green) within islet cells with membrane labeled by *neurod:memKate* **(A, magenta)** or cytoplasmic labeling by *pax6b:dsRed* **(B, magenta)**. Mitochondria within protrusions are found as discrete units separated from (blue arrowheads) or extending from (yellow arrowheads) the cell body.

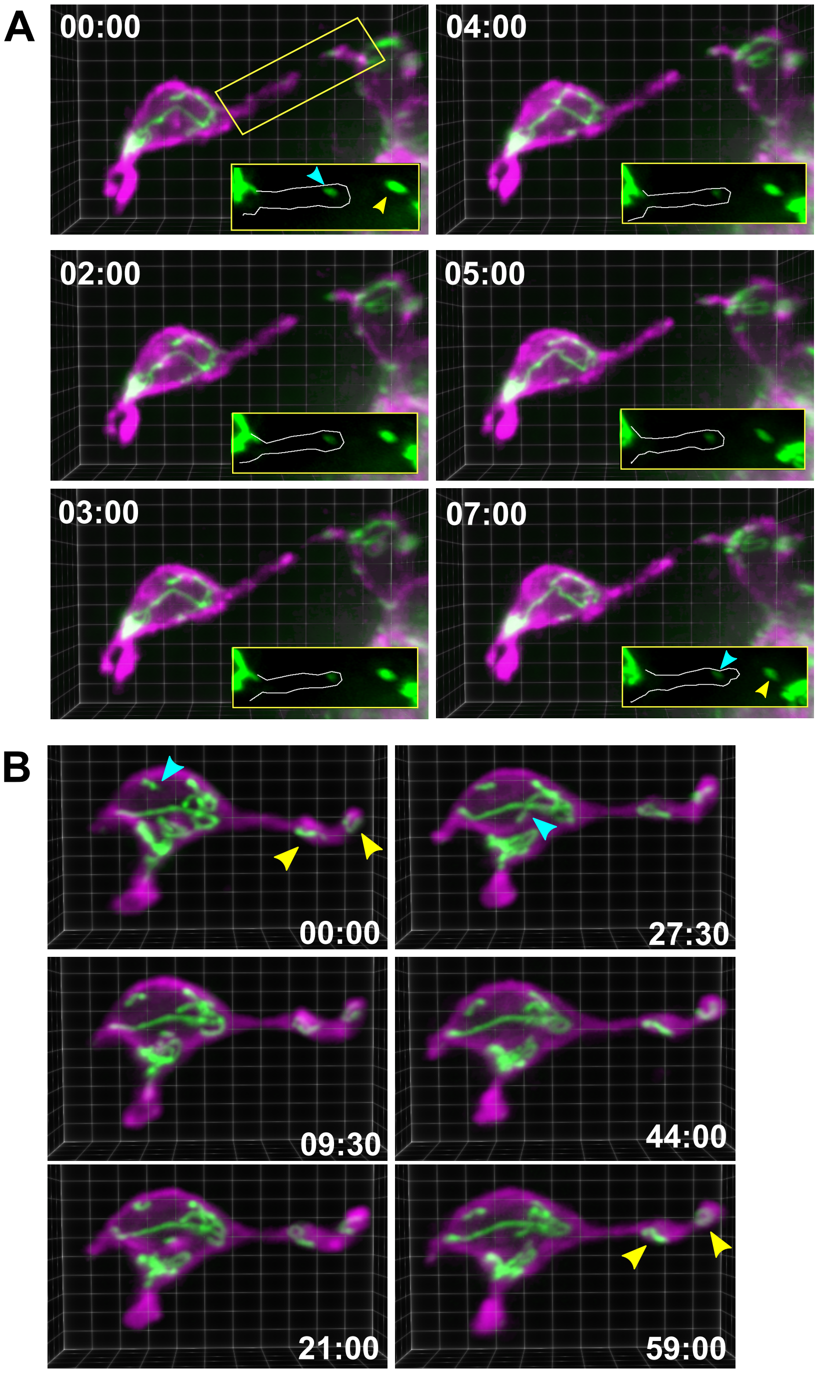

Supplementary Figure 7. Dynamic mitochondrial behavior captured in time lapse images. 3D visualizations of selected time points from *in vivo* time lapse imaging. Islet cell mitochondria are labeled with *neurod:mito-GFP* (green) and cell membranes with *neurod:memKate* (magenta). **(A)** Small mitochondria are found within the tip region of interacting cell protrusions (arrowheads). Inset (yellow box) shows GFP channel alone to expose mitochondria within the protrusions (white outline). Time points as indicated (min:sec). (See also **Video 2**.) **(B)** Mitochondria within the cell body show extension, retraction and translocations (blue arrowheads). Mitochondria within protrusions change shape but are maintained at a fixed location (yellow arrowheads).

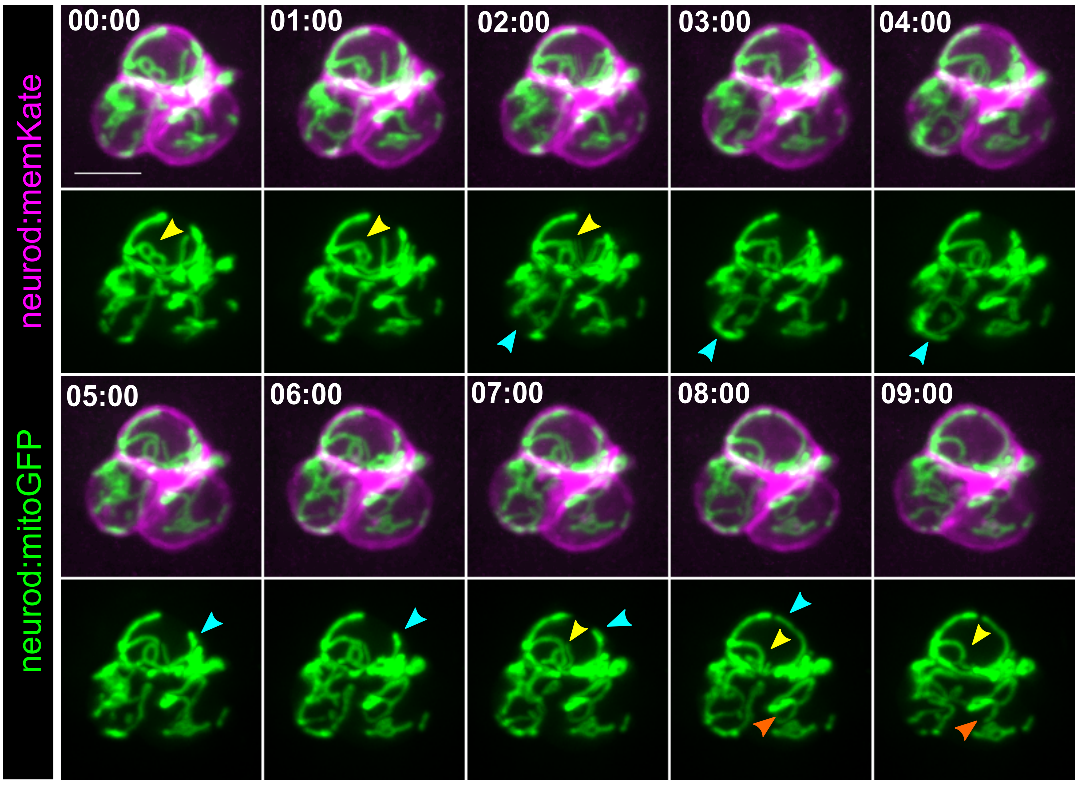

Supplementary Figure 8. Mitochondrial motility in clustered cells.
3D visualizations of selected time points from *in vivo* time lapse imaging of a small cell cluster. Islet cell mitochondria are labeled with *neurod:mito-GFP* (green) and cell membranes with *neurod:memKate* (magenta). Mitochondria at the periphery extend and retract (blue arrowheads), loops and short fragments with the cell move and change shape (yellow, orange arrowheads). (See also **Video 3**.)

Supplementary Information

Supplementary Videos

Video 1

Time lapse movie with image capture at the indicated times. 3D visualization of isolated islet cell expressing *neurod:mito-GFP* (mitochondria, green) and *neurod:memKate* (cell membranes, magenta) Related to Fig **5A**.

Video 2

Time lapse movie with image capture at the indicated times. 3D visualization of isolated islet cell expressing *neurod:mito-GFP* (mitochondria, green) and *neurod:memKate* (cell membranes, magenta). Related to Fig **S7B**.

Video 3

Time lapse movie with image capture at the indicated times. 3D visualization of a small endocrine cell cluster showing (left) labeled mitochondria (green, *neurod:mito-GFP*) and cell membranes (magenta, *neurod:memKate*), GFP channel alone for clarity (right). Related to Fig **S8**.

Supplementary Methods

Optimization of MitoGraph analysis

Based on visual assessment comparing the segmentation .png image to the original data, the following options may be applied to improve the outcome: (1) Deconvolution prior to analysis can enhance resolution and reduce noise, but this may result in the removal of weak mitochondrial signals. (2) When images are cropped using an irregularly-shaped region of interest (ROI), Gaussian noise may be applied external to the ROI to correct spurious mitochondria signals [1]. (3) The 'scales' parameter can be adjusted to compensate for low signal to noise ratio [2]. In cases where segmentation is largely accurate, but with minor deviations, incorrectly detected fragments can be manually removed based on identification of the involved nodes in Paraview.

Separation of mitochondria in adjacent cells (Blue text indicates Fiji macro language)

Requires: 3D ImageJ Suite (https://github.com/mcib3d/mcib3d-plugins [3].

To separate mitochondria contained within adjacent cells, the following steps were applied in Fiji:

1. Mask file (.nii from MITK) is opened in Fiji. Set x-y-z stack dimensions.

2. Mask is smoothed using '3D Binary Close Labels' (3D ImageJ Suite).

```
run("3D Binary Close Labels", "radiusxy=1 radiusz=1");
```

3. Mask (z-stack) is applied to corresponding mitoGFP image stack ("Image") using Image Calculator.

```
imageCalculator("Multiply create stack", "Image"," CloseLabels");
```

4. Save as ‘MaskedCell’.

Addition of Gaussian noise external to cell mask (*Blue text indicates Fiji macro language****)***

To improve segmentation by adding simulated background noise:

1. Make blank stack to match image stack (use x, y, z dimensions)

```
newImage("HyperStack", "16-bit grayscale-mode", width, height, 1, slices, 1);
```

2. Add noise to stack

```
run("Add...", "value=400 stack");
run("Add Specified Noise...", "stack standard=35");
rename("Hyperstack_Noise");
```

3. Open mask file (“Cell_Mask.nii”). Set x-y-z stack dimensions.

4. Smooth mask using ‘3D Binary Close Labels’ (3D ImageJ Suite).

```
run("3D Binary Close Labels", "radiusxy=1 radiusz=1");
```

5. Invert signal of mask (check values: 0 inside object, 1 background)

```
run("Invert", "stack");
```

6. Multiply inverted mask x noise hyperstack

```
imageCalculator("Multiply create stack", "Cell_Mask","Hyperstack_Noise");
rename("NoiseMask");
```

7. Add mask with noise to masked cell (Open file ‘MaskedCell’).

```
imageCalculator("Add create stack", "MaskedCell","NoiseMask");
```

The resulting stack can be analyzed in MitoGraph.

Processing of Time Lapse Series

Regions of interest containing single cells or clusters from 5D (XYZCT) stacks were processed as follows: (1) The *neurod:memKate* channel was denoised with Noise2Void [4]. (2) The *neurod:mitoGFP* signal was aligned within a single z-stack to correct for jitter movements, and the transformation applied to the memKate channel using the MultiStackReg plugin. (3) z-stacks were aligned between time-points using the Correct 3D Drift plugin [5]. For preparation of time lapse figures, the following steps were performed as required: Unsharp Mask and bleach correction were applied to the mitoGFP channel, and brightness and contrast were uniformly adjusted. For 3D visualizations, images were rescaled 2-fold along the z-axis, with preservation of image dimensions, to improve visibility of cell boundaries and fine protrusions. 3D visualizations were prepared using the Fiji 3D Viewer or ClearVolume [6].

Supplementary References

1. Viana, M. P., Brown, A. I., Mueller, I. A., Goul, C., Koslover, E. F. & Rafelski, S. M. (2020) Mitochondrial Fission and Fusion Dynamics Generate Efficient, Robust, and Evenly Distributed Network Topologies in Budding Yeast Cells, *Cell Syst.* **10**, 287-297 e5.
2. Harwig, M. C., Viana, M. P., Egner, J. M., Harwig, J. J., Widlansky, M. E., Rafelski, S. M. & Hill, R. B. (2018) Methods for imaging mammalian mitochondrial morphology: A prospective on MitoGraph, *Anal Biochem.* **552**, 81-99.
3. Ollion, J., Cochennec, J., Loll, F., Escude, C. & Boudier, T. (2013) TANGO: a generic tool for high-throughput 3D image analysis for studying nuclear organization, *Bioinformatics.* **29**, 1840-1.
4. Krull, A. B., Tim-Oliver; Jug, Florian (2019) Noise2void-learning denoising from single noisy images in *Proceedings of the IEEE Conference on Computer Vision and Pattern Recognition* pp. 2129--2137.
5. Parslow, A., Cardona, A. & Bryson-Richardson, R. J. (2014) Sample drift correction following 4D confocal time-lapse imaging, *J Vis Exp*, 86.
6. Royer, L. A., Weigert, M., Gunther, U., Maghelli, N., Jug, F., Sbalzarini, I. F. & Myers, E. W. (2015) ClearVolume: open-source live 3D visualization for light-sheet microscopy, *Nature methods.* **12**, 480-1.
